# Study of Hepatitis E Virus-4 Infection in Human Liver-Chimeric, Immunodeficient, and Immunocompetent Mice

**DOI:** 10.3389/fmicb.2022.819877

**Published:** 2022-02-28

**Authors:** Laura Collignon, Lieven Verhoye, Renate Hakze-Van der Honing, Wim H. M. Van der Poel, Philip Meuleman

**Affiliations:** ^1^Laboratory of Liver Infectious Diseases, Department of Diagnostic Sciences, Faculty of Medicine and Health Sciences, Ghent University, Ghent, Belgium; ^2^Wageningen Bioveterinary Research, Wageningen University and Research, Lelystad, Netherlands

**Keywords:** hepatitis E virus, human liver chimeric mice, immunocompetent mouse model, viral tropism, immune deficiency, subtype

## Abstract

The hepatitis E virus (HEV) is responsible for 20 million infections worldwide per year. Although, HEV infection is mostly self-limiting, immunocompromised individuals may evolve toward chronicity. The lack of an efficient small animal model has hampered the study of HEV and the discovery of anti-HEV therapies. Furthermore, new HEV strains, infectious to humans, are being discovered. Human liver-chimeric mice have greatly aided in the understanding of HEV, but only two genotypes (HEV-1 and HEV-3) have been studied in this model. Moreover, the immunodeficient nature of this mouse model does not allow full investigation of the virus and all aspects of its interaction with the host. Recent studies have shown the susceptibility of regular and nude Balb/c mice to a HEV-4 strain (KM01). This model should allow the investigation of the interplay between HEV and the adaptive immune system of its host, and potential immune-mediated complications. Here, we assess the susceptibility of human liver-chimeric and non-humanised mice to a different HEV-4 strain (BeSW67HEV4-2008). We report that humanised mice could be readily infected with this isolate, resulting in an infection pattern comparable to HEV-3 infection. Despite these results and in contrast to KM01, non-humanised mice were not susceptible to infection with this viral strain. Further investigation, using other HEV-4 isolates, is needed to conclusively determine HEV-4 tropism and mouse susceptibility.

## Introduction

The single-stranded RNA virus, hepatitis E virus (HEV) is enterically transmitted and is annually responsible for an estimated 20 million infections worldwide (WHO, 2021). HEV belongs to the *Hepeviridae* family, where viral strains, known to infect humans, are classified in the *Orthohepevirus A* species. Most HEV infections occur in low- to middle-income countries in Asia, Africa, and Latin America, where HEV is spread *via* the faecal-oral route. Here, the transmission is instigated by poor sanitary infrastructure and hygienic practises, which results in sporadic cases and occasionally in large outbreaks. In these regions, HEV genotype 1 (HEV-1) and HEV-2 are most commonly detected (Sayed et al., 2015; European Association for the Study of the Liver [EASL], 2018). Contrary to previous belief, hepatitis E is not a disease confined to developing countries or travellers returning from such regions. Autochthonous hepatitis E is an emerging problem across Europe, North America, New Zealand, and Japan. Here, the prevalent strains, HEV-3 and HEV-4, cause zoonotic infections in humans and usually present as single infections after consumption of contaminated animal products. Although HEV infects a broad range of species, including bats, ferrets, rabbits and chickens, the primary species that are considered reservoirs for transmission to humans are pigs, deer and wild boar (Kamar et al., 2012). Furthermore, transfusion-transmitted HEV infection has been described. A study in the United Kingdom involving 225,000 blood donations showed that 0.035% of these donations were viremic (Hewitt et al., 2014). Also, screening of 38,137 Flemish blood donations showed 0.018% to be viremic (Vercouter et al., 2019). Hewitt et al. (2014) demonstrated a viral transmission rate of 42% to recipients of HEV-containing blood products.

Although, acute HEV infections are mostly self-limiting without overt disease, severe complications may be seen in pregnant women, infants and patients with pre-existing liver disease. Moreover, chronic hepatitis E cases have been reported for HEV-3 and HEV-4 in immunocompromised individuals (solid organ transplant recipients, patients with haematological disorders or infected with HIV) and extrahepatic manifestations are increasingly recognised (e.g., neurological and renal injury, and haematological disorders) (Kamar et al., 2014). Ribavirin is the treatment of choice for patients with chronic HEV infections, but this is an off-label use and HEV strains with reduced sensitivity to ribavirin are being reported with increasing frequency (Donnelly et al., 2017; European Association for the Study of the Liver [EASL], 2018). Therefore, the need for direct-acting anti-viral therapies arises.

The study of HEV has long been hampered due to the lack of efficient cell culture and animal models. Although, several viral strains of HEV-3 and HEV-4 have been adapted to grow efficiently in cell culture, viruses of HEV-1 still replicate poorly *in vitro* (Shukla et al., 2011). Also, a robust small animal model for the study of HEV infection is still lacking. Chimpanzees, cynomolgus, and rhesus macaques are susceptible to infection with HEV-1 to HEV-4, but clinical presentations of disease are sometimes limited and chronic HEV infection has not yet been described in non-human primates. Also, due to ethical concerns, the use of chimpanzees is now severely restricted (Sayed et al., 2017b; Corneillie et al., 2019). As a natural reservoir of HEV-3 and HEV-4, pigs serve as an important homologous animal model system and have aided in a better understanding of HEV infection. Furthermore, extrahepatic replication of HEV has been reported as well as a pig model mimicking chronic HEV infection by administration of immunosuppressive drugs (Williams et al., 2001; Cao et al., 2017; Corneillie et al., 2019). However, pigs are not susceptible to HEV-1 and HEV-2; and infection does not result in observable clinical disease (Bouwknegt et al., 2009; Corneillie et al., 2019). Other small animal models have been investigated, including rabbits, rats and mice, to study pathogenesis and identify new anti-viral compounds. Also, animal models are used to study the effect of HEV infection on certain subpopulations like pregnant women and immunocompromised patients.

Successful HEV infections have been established in human liver-chimeric mouse models (e.g., uPA^+/+^-SCID, uPA^+/+^-NOG, FRG) with HEV-1 and HEV-3 (van de Garde et al., 2016; Sayed et al., 2017a,b). These mice can be chronically infected, with consistent detection of HEV RNA and HEV open reading frame (ORF)-2 Ag for up to 16 weeks post inoculation. Although human liver-chimeric mouse models have greatly aided in the understanding of HEV pathogenesis and the evaluation of new anti-HEV therapies, the immunodeficient nature of these models do not allow the study of the interplay between HEV and the adaptive host immune system. Furthermore, their susceptibility to other human isolates (HEV-2, HEV-4, and HEV-7) has not been studied yet (Sayed and Meuleman, 2020). Next to human liver-chimeric mice, numerous studies have attempted to infect non-humanised mice with HEV. It was reported that wild-type and immunodeficient mice were not susceptible to human-derived HEV-1, human-derived HEV-3, wild boar-derived HEV-3 and HEV-4 (Li et al., 2008; Schlosser et al., 2014; Sayed and Meuleman, 2017; Sayed et al., 2017a,b). However, Balb/c and Balb/c nude mice could be infected by HEV-4 isolated from a pig stool isolated in China (Zhou et al., 2017; Yang et al., 2019). HEV RNA and HEV Ag were detected in liver, spleen, kidney, brain, uterus and intestine of infected mice. Importantly, in these studies only one HEV-4 isolate (KM01) was infectious to non-humanised mice. A recent study of Li et al. (2020) extended these results by identifying eight more HEV-4 viral strains, infectious to Balb/c mice. These strains were human- and cow-derived, and together with KM01 belong to the HEV-4h subtype. Furthermore, this study revealed a discrepancy between Balb/c and C57BL/6 mice, where Balb/c, but not C57BL/6 mice, were susceptible to HEV-4 infection (Li et al., 2020). Specific host factors and differences in immunological status may contribute to this discrepancy. For example, one of the major differences between Balb/c and C57BL/6 mice is the bias toward a T helper cell (Th)-2 versus a Th1 response upon infection, respectively. In general, Th1 cells are important in the clearance of intracellular pathogens, whereas Th2 cells are commonly associated with responses against parasitic infections (Sellers et al., 2012).

In this study, we investigated the susceptibility of human liver-chimeric and non-humanised mice to another pig-derived HEV-4 strain (BeSW67HEV4-2008) (Hakze-van der Honing et al., 2011). Different immunocompetent and immunocompromised mouse strains were chosen, based on their immunological profile. We report that human liver-chimeric mice can readily be infected with this strain, whereas the infection in non-humanised mice did not result in detection of HEV RNA in faeces or organs (liver, spleen, kidney, intestine, brain, uterus) collected from these mice.

## Materials and Methods

### Animals and Viruses

Human liver-chimeric mice were produced as previously described (Meuleman et al., 2005). Briefly, homozygous uPA^+/+^-SCID mice were transplanted with 0.7 × 10^6^ cryopreserved primary human hepatocytes (donor C342, Corning or HUM191501, Lonza), *via* intrasplenic injection. Human albumin concentration in plasma was determined 6 weeks after transplantation by ELISA (Bethyl Laboratories, Montgomery, Texas, United States) and was used as a marker of liver chimerism. SCID mice originated from the uPA-SCID breeding colony (uPA^–/–^-SCID offspring) and had a Balb/c background. Balb/cAnNRj, Balb/cJRj, C57BL/6NRj, DBA/2, and SJL mice (female, 6–8 weeks old) were purchased from Janvier Labs (Le Genest-Saint-Isle, France). All mice were bred and/or handled under specific pathogen free conditions in the animal facility of the Faculty of Medicine and Health Sciences, Ghent University.

The HEV-1 viral strain (SAR-55, stool suspension from an infected chimpanzee, GenBank accession no. M80581) as well as the HEV-4 strain (BeSW67HEV4-2008, stool suspension from an infected pig, GenBank accession no. HQ842727) were passaged through human liver-chimeric mice by inoculating the mice with a 10% (w/v) stool suspension. Of these infected mice, faecal samples were collected and 10% (w/v) stool suspensions were made, filtered through a 0.22 μm filter and used to inoculate human liver-chimeric and non-humanised mice. Mice were inoculated *via* intrasplenic or intraperitoneal injection or *via* oral gavage with a viral dose of 10^6^ IU/mouse or as otherwise stated. Faecal and EDTA-plasma samples were regularly collected for analysis. Upon humane euthanasia, different organs (liver, spleen, kidney, intestine brain and uterus) were collected and preserved in RNAlater (Merck, Overijse, Belgium). The animal study protocol was approved by the Animal Ethics Committee of the Faculty of Medicine and Health Sciences of Ghent University.

### Quantification of Hepatitis E Virus RNA

Total RNA was extracted from EDTA-plasma or 10% (w/v) stool suspensions using the NucliSENS easyMAG system (Biomérieux, Craponne, France). Following extraction, a one-step real-time RT-qPCR reaction was performed using the LightCycler 480 (Roche Diagnostics, Belgium), the LightCycler multiplex RNA virus Master mix and following primers and probe targeting the ORF2/ORF3 overlapping region: forward primer: 5′-GGTGGTTTCTGGGGTGAC-3′, reverse primer: 5′-AGGGGTTGGTTGGATGAA-3′ and probe: 5′-FAM-TGATTCTCAGCCCTTCGC-TAMRA-3′ (Jothikumar et al., 2006; Abravanel et al., 2012). A standard curve was generated and calibrated against the WHO 1st international standard of HEV (Paul-Ehrlich Institute, Germany) (Baylis et al., 2013). The limit of detection (LOD) of this assay on undiluted samples was 40 IU/ml and equals the 100% limit of quantification (LOQ). However, due to dilution of the mouse samples the actual LOD may be higher and is indicated at each graph.

For total RNA extraction from tissues, the AllPrep RNA/DNA/protein kit (Qiagen, Venlo, Netherlands) was used according to manufacturer’s instructions. HEV RNA was quantified in total tissue RNA by RT-qPCR as described above.

### Detection of Anti-Hepatitis E Virus ORF2 Antibodies

For this assay, a 6×His-tagged HEV ORF2 protein was constructed based on the SAR-55 strain (HEV-1) sequence. This protein was expressed in CHO cells and purified using the HisTrap*™* High Performance column (Merck). For the detection of anti-HEV ORF2 antibodies, the purified 6×His-tagged HEV ORF2 protein was diluted in carbonate buffer to a concentration of 2 μg/ml and coated by overnight incubation at 4°C on Nunc Maxisorp plates (Thermo Fisher Scientific, Merelbeke, Belgium). Unbound protein was washed away by 0.05% Tween-20 in PBS and plates were blocked with 0.5% milk powder and 5% goat serum in wash buffer for 1 h at room temperature. Subsequently, mouse EDTA-plasma samples were added and incubated for 2 h at room temperature. Plates were washed and a polyclonal horseradish peroxidase-conjugated sheep anti-mouse immunoglobulin antibody (Merck), diluted 1/1.000 in blocking buffer, was added. The plates were incubated for 1 h at room temperature and washed. 3,3′,5,5′-Tetramethylbenzidine (TMB, Merck) was added and incubated for 15 min at room temperature in the dark. Colour development was stopped with 1N H_2_SO_4_ and absorbance at 450 and 650 nm (reference wavelength) was measured. Final OD values are calculated by subtraction of the OD_650nm_ from the OD_450nm_ and the cut-off value is based on the OD value of PBS-inoculated mice (mean + 3 standard deviations). Results are represented as signal to cut-off values, where values above one are considered positive.

### Whole Genome Sequencing

To allow whole genome sequencing, primers were designed based on the alignment of 20 HEV-4 sequences, including reference sequences described by Smith et al. (2020). Seven primer sets were used to amplify the viral genome and these primers contained a M13 tail to enable Sanger sequencing. Next to M13 primers, additional primers were designed for whole genome sequencing (Table 1).

**TABLE 1:** PCR and sequencing primers for full genome sequencing of HEV-4.

Primer name	Primer sequence (5′-3′)	Nucleotide position
HEV4-WGA-1F^a^	**TGTAAAACGACGGCCAGT**GGCA GACCACGTATGTGGTCGAC	1–23
HEV4-WGA-1R^a^	**CAGGAAACAGCTATGACC**RGARA TACTSACRGTNGCCTCC	1559–1582
HEV4-WGA-2F^b^	**TGTAAAACGACGGCCAGT**ACTCTA AGGGGATGAAGAGGCTGGAG	1199–1223
HEV4-WGA-2R^b^	**CAGGAAACAGCTATGACC**AGTCT CCCGATAAGCAGCTTCAAGC	2657–2680
HEV4-WGA-3F^a^	**TGTAAAACGACGGCCAGT**CAYAGYT GGGAGTCTGCTAACC	2085–2103
HEV4-WGA-3R^a^	**CAGGAAACAGCTATGACC**ATRCCR ACCTCACGRAGGAG	3624–3643
HEV4-WGA-4F^c^	**TGTAAAACGACGGCCAGT**GGCCGT AGGGTTGTTATTGATGAGG	3124–3150
HEV4-WGA-4R^c^	**CAGGAAACAGCTATGACC**GGAC TCYTTYGGGGCCTG	4566–4583
HEV4-WGA-5F^b^	**TGTAAAACGACGGCCAGT**ACCATG CAGATGTTCGTGGGTCTC	4028–4050
HEV4-WGA-5R^b^	**CAGGAAACAGCTATGACC**ACGAG AATCGACATCAGGAACTG	5543–5568
HEV4-WGA-6F^b^	**TGTAAAACGACGGCCAGT**ATGCG CTCTCGGGCTCTTC	5176–5200
HEV4-WGA-6R^b^	**CAGGAAACAGCTATGACC**GCTGTAA GTGAAAGCCAAAGCACATCG	6585–6611
HEV4-WGA-7F^c^	**TGTAAAACGACGGCCAGT**TAACTAC TACTGCTGCYACACGYTTTA	6205–6229
HEV4-WGA-7R^c^	**CAGGAAACAGCTATGACC**GGAG CGCGRAACGCAGAARA	7210–7229
HEV4-WGS-1F	CAGACCACGTATGTGGTC	3–20
HEV4-WGS-1R	CGAGGTGGTGTGGTAAGTAC	628–646
HEV4-WGS-2F	GRCTTCCACCTGCTGAYC	436–453
HEV4-WGS-2R	CAACYGTGACCTTGTAACTG	1067–1086
HEV4-WGS-3F	ATGTTCCATACCCTCGYTCKAC	859–880
HEV4-WGS-3R	AGGGTCAACATCCGANCC	1539–1553
HEV4-WGS-4F	TCTGCCGGCTTYCAYCTYGAC	1341–1361
HEV4-WGS-4R	ATAAGAGAATGCCKCTGRRCAC	1999–2020
HEV4-WGS-5F	YTGTCATTTGAYCCGGCNAAGC	1800–1821
HEV4-WGS-5R	CATTCAGACTCAAAMAGAGAGC	2440–2461
HEV4-WGS-6F	TGTTTYTCCCCACTYGAG	2163–2180
HEV4-WGS-6R	AGGTARGGTAGGTCGGTTRG	2830–2849
HEV4-WGS-7F	TTGAGGCCGCYTATCGGGAGAC	2659–2680
HEV4-WGS-7R	GGCATCTRTGGGTRAGRTGC	3305–3324
HEV4-WGS-8F	YAGGGTTGTTATTGATGAGG	3131–3150
HEV4-WGS-8R	TCAGCCAGCTGGTGATAAG	3781–3799
HEV4-WGS-9F	CACACNGRRAARTGTGTGG	3588–3606
HEV4-WGS-9R	TACCATGCGCTATCGTCTCACC	4230–4251
HEV4-WGS-10F	AAGYTGTAYGARGCNGCCCATG	4011–4032
HEV4-WGS-10R	AGAATCATCCCCTTTRAAYG	4699–4718
HEV4-WGS-11F	TYTCAYTNGGGTTGGAGTG	4471–4489
HEV4-WGS-11R	GCTAGTAAGGTCAAGAACAGG	5097–5116
HEV4-WGS-12F	AGCTNCGGCTGGCWGTYTGTG	4927–4947
HEV4-WGS-12R	YTGACGGCGCAASATAGCACC	5566–5586
HEV4-WGS-13F	CCTATATTCATCCAACCAACC	5342–5362
HEV4-WGS-13R	GGAATTTACAGGTGATCCATG	6022–6042
HEV4-WGS-14F	GACNTCTGTTGATATGAAYTC	5838–5858
HEV4-WGS-14R	GACGGRGTNGGACGRTCTTG	6526–6545
HEV4-WGS-15F	AATCTTGCTGATACGCTTCTCG	6301–6322
HEV4-WGS-15R	CCCAGAACCYAAATTAGTAGTG	6933–6954
HEV4-WGS-16F	YAAGGTCACYCTTGATGG	6732–6749
HEV4-WGS-16R	GCGAAACGCAGAAGAGAG	7208–7224
HEV4-WGS-17F	CARTCWACTGTYGCTGAGCNC	7102–7119

*M13F and M13R primer sequences are highlighted in bold. WGA, whole-genome amplification; WGS, whole-genome sequencing; F, forward; R, reverse. (a) Ta = 64°C, (b) Ta = 71°C, and (c) Ta = 67°C.*

HEV-4 RNA was extracted from pig-derived and mouse-passaged 10% (w/v) stool suspensions, using the NucliSENS easyMAG system. cDNA synthesis was performed with the SuperScript IV VILO mastermix with ezDNase Enzyme (Thermo Fisher Scientific), according to manufacturer’s instructions. Genome fragments were amplified by PCR with a mixture (50 μl) containing cDNA, 1× Q5 reaction buffer, 1× Q5 High GC Enhancer, 200 μM of each dNTP, 0.5 μM of forward, and reverse primer and 1 unit of Q5 High-Fidelity DNA polymerase (New England Biolabs, Bioké, Leiden, Netherlands). Cycling conditions are as follows: 98°C for 30 s followed by 35 cycli of 98°C for 10 s, Ta for 30 s, 72°C for 45 s; 72°C for 2 min (Ta is specified in Table 1). All PCR products were purified with the Minelute PCR purification kit (Qiagen) and sent to Eurofins – GATC Biotech (Germany) for Sanger sequencing. The resulting sequences were assembled with the CLC main workbench 7 software.

### Phylogenetic Analysis

Sequences of the SAR-55 and KM01 strains were retrieved from GenBank along with HEV reference sequences of HEV-4 as described by Smith et al. (2020) Reference sequences of HEV-1 and HEV-3 were included as outgroup for the analysis. Together with the sequences of the pig-derived and mouse-passaged HEV-4, sequences were aligned with Muscle. A statistical selection of the best-fit model of nucleotide substitution was performed using the corrected Akaike information criterion (AICc). A maximum likelihood phylogenetic tree was constructed based on the General Time Reversible and Gamma distribution with Invariant Sites (GTR + G + I) model with 1,000 bootstrap replicas. Analyses and tree construction were performed using the Mega X software.

### Statistical Analysis

Statistical analysis was performed with the GraphPad Prism 7.0. software. Statistical significance of differences between groups was tested with the Mann-Whitney *U* test. A probability value of *p* < 0.05 was considered significant.

## Results

### Hepatitis E Virus-4 Infection in Human Liver-Chimeric Mice

**FIGURE 1:**
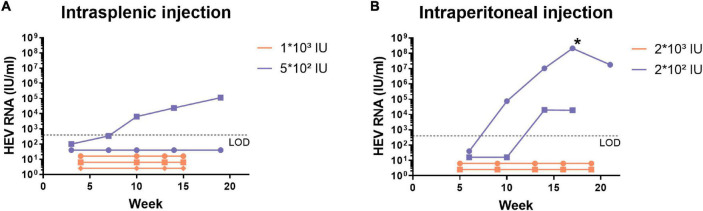
Human liver-chimeric mice can be infected with the pig-derived HEV-4 strain BeSW67HEV4-2008 after intrasplenic or intraperitoneal injection. Human liver-chimeric mice were inoculated *via* intrasplenic **(A)** or intraperitoneal **(B)** injection with different viral doses of the BeSW67HEV4-2008 HEV-4 strain. Viral RNA was quantified in 10% (w/v) stool samples, collected at different time points. The asterisk (*) represents the time point of which the mouse-passaged HEV-4 10% (w/v) stool suspension was prepared. LOD, limit of detection.

We investigated the susceptibility of the humanised uPA^+/+^-SCID mouse model to the pig-derived HEV-4 viral isolate BeSW67HEV4-2008 (Hakze-van der Honing et al., 2011). A 10% (w/v) stool suspension, containing the HEV-4 strain, was used to inoculate humanised uPA^+/+^-SCID mice by intrasplenic or intraperitoneal injection and with different doses ranging from 2 × 10^2^ to 2 × 10^3^ IU. Faecal and blood samples were taken at regular intervals and tested for the presence of HEV RNA (Figure 1). Of the nine inoculated mice, only three mice showed signs of infection after 10 to 14 weeks post-inoculation. In one mouse, which was injected intraperitoneally, the HEV RNA faecal titers rose as high as 10^8^ IU/ml after 17 weeks of infection (Figure 1, marked with *). A 10% (w/v) stool suspension was made from faecal samples, collected at this time point and this mouse-passaged HEV-4 suspension was used in all subsequent experiments.

**FIGURE 2:**
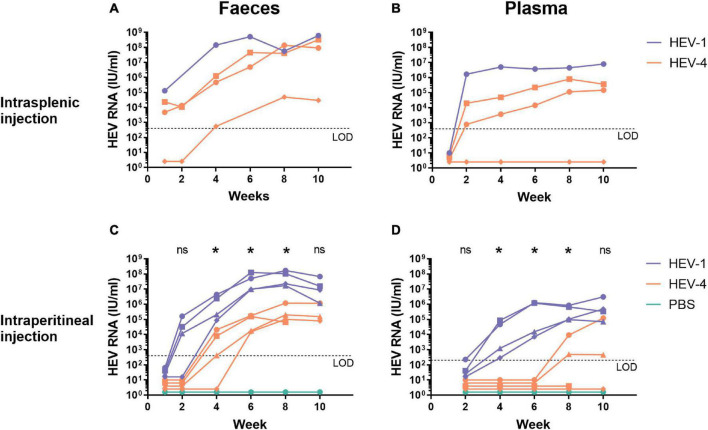
Human liver-chimeric mice can be readily infected with the mouse-passaged faecal suspension of the HEV-4 strain BeSW67HEV4-2008 as well as the HEV-1 strain SAR-55 *via* intrasplenic or intraperitoneal injection. A 10% (w/v) stool suspension was prepared from a HEV-4 (BeSW67HEV4-2008) or HEV-1 (SAR-55) infected humanised mouse and used to inoculate naïve human liver-chimeric mice. Naïve mice were intrasplenically **(A,B)** or intraperitoneally **(C,D)** injected with 10^6^ IU HEV RNA. At different time points, faeces [10% (w/v) suspensions, **A,C**] and plasma **(B,D)** samples were collected and analysed for the presence of HEV RNA. Different symbols are used to distinguish between individual mice and allow the comparison of corresponding faecal and plasma data of a single animal. The asterisk (*) indicates there is a significant difference between HEV-1 and HEV-4 at that time point (*p* < 0.03). LOD, limit of detection; PBS, phosphate buffered saline; ns, not significant.

After this first successful establishment of HEV-4 infection, we further characterised HEV-4 infection in human liver-chimeric mice. During these experiments, HEV-4 infection was compared to HEV-1 (SAR-55 strain) infection, which has been extensively studied in this model (Sayed et al., 2017b). Humanised mice were inoculated *via* intrasplenic or intraperitoneal injection with the same viral dose of HEV-1 or HEV-4 (10^6^ IU/mouse). At regular time points, faecal, and blood samples were collected for analysis (Figure 2). All three mice, intrasplenically inoculated with HEV-4, became infected. For two out of three mice, HEV RNA was detectable in stool as soon as one week post-inoculation and the viral titer increased with time to levels reaching 10^8^ IU/ml (Figure 2A). In plasma, viral titers were only detected after 2 weeks of infection and reached up to 10^5^ IU/ml (Figure 2B). Also, viral titers in plasma seemed to reach a plateau, whereas stool titers steadily increased over time. This was all in line with HEV-1 infection, although the plasma titer was 10-fold higher in HEV-1 infected mice compared to HEV-4. The third mice in the HEV-4 group showed a considerable delay in the onset of infection. HEV RNA was detected in faeces from week 4 post-inoculation onward, however, viral RNA was never detected in plasma. At initiation of this experiment, this mouse had the lowest concentration of human albumin in its plasma (3.8 mg/ml compared to 6.2 and 5.6 mg/ml). Less extensive liver chimerism may have contributed to the slower viral kinetics and lower infection titers observed.

After intraperitoneal inoculation, the onset of infection is delayed for both HEV-1 and HEV-4 compared to intrasplenic injection (Figures 2C,D). Still, viral titers of HEV-1 can be detected in stool from week 2 onward, whereas HEV-4 was only detectable in faeces after four to six weeks post-inoculation. As for the HEV titers in plasma, two out of four mice, injected with HEV-4, showed positive titers after eight weeks of infection and in the other two mice, HEV RNA was never detected in plasma. In contrast, in all mice, injected with HEV-1, HEV RNA was detected in plasma after four weeks of infection. Also, a decline in faecal and plasma titers was observed in some HEV-1 infected mice after ten weeks of infection. This decline coincided with a decrease in human albumin levels, indicating spontaneous decline of the human liver graft. Statistical analyses, comparing HEV-1 and HEV-4 infected mice per collection point, showed HEV-1 titers in stool and plasma were significantly higher from week 4 to week 8 of infection (Figures 2C,D). Whole genome sequencing of the original pig-derived and the mouse-passaged viral stock revealed one point mutation (T3155C) in ORF1, which did not result in any amino acid changes.

### Hepatitis E Virus-4 Infection in Non-humanised Mice

Based on the work of Li et al. (2020) we attempted to corroborate HEV-4 infection in non-humanised mice with another pig-derived HEV-4 strain, BeSW67HEV4-2008 (Hakze-van der Honing et al., 2011). As inoculum we utilised the same mouse-passaged HEV-4 preparation that proved infectious in humanised mice, and as reference we again used the HEV-1 strain SAR-55. Initially, we compared HEV infection in Balb/cAnNRj, C57BL/6NRj and SCID mice. For each mouse strains, 10 mice were randomly divided into three groups. Group 1 (*n* = 4) was injected with HEV-4, group 2 (*n* = 4) was injected with HEV-1 and group 3 (*n* = 2) was injected with phosphate buffered saline (PBS) and served as a negative control. All mice were intraperitoneally inoculated with the same viral dose as used in experiments with humanised mice (10^6^ IU/mouse). Faecal and blood samples were collected for ten weeks, after which the mice were humanely euthanised and different organs were collected for further analysis. Stool samples, collected at week 4 and 8 post-inoculation, were subjected to RT-qPCR to determine the presence of HEV RNA, but no viral RNA was detected (data not shown). Furthermore, total RNA was extracted from different organs, collected from these animals (liver, spleen, kidney, intestine, brain and uterus) and analysed for the presence of HEV RNA. However, none of the analysed organs, showed the presence of HEV RNA (data not shown).

Subsequently, additional mouse strains (DBA/2, SJL, and Balb/cJRj) were examined for their susceptibility to HEV-1 or HEV-4 infection. Identical to the previous experiments, ten mice were grouped and inoculated with HEV-1, HEV-4, or PBS. Similar to previous experiments, HEV RNA could not be detected in stool samples collected from these mice, nor in their liver (data not shown).

### Alternative Inoculation Routes for the Infection of Non-humanised Mice

**FIGURE 3:**
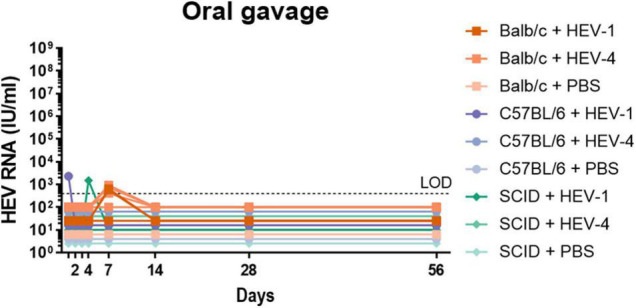
Non-humanised mice are not susceptible to infection with HEV-4 strain BeSW67HEV4-2008 or HEV-1 strain SAR-55 *via* oral inoculation. A humanised mouse-passaged 10% (w/v) stool suspension of a HEV-4 (BeSW67HEV4-2008) or HEV-1 (SAR-55) strain were used to inoculate Balb/c, C57BL/6 or SCID mice. All mice were inoculated with 10^6^ IU HEV RNA *via* oral gavage and stool samples were collected at different time points. Ten percent (w/v) stool suspensions of these samples were subjected to HEV RT-qPCR. LOD, limit of detection; PBS, phosphate buffered saline.

In order to determine the role of the inoculation route in the establishment of HEV infection, two other infection routes were investigated: intrasplenic injection and oral gavage. Oral gavage is the most natural infection route, whereas intrasplenic injection is the most direct route to the liver. Again, Balb/cAnNRj, C57BL/6NRj and SCID mice were randomly divided into three groups; inoculated with HEV-1, HEV-4 or PBS and monitored for ten weeks. Identical to intraperitoneal injection, no HEV RNA could be detected in intrasplenically injected mice at week 4 and 8 post-inoculation (data not shown). Furthermore, viral RNA could not be detected in the organs of these mice (data not shown). Additional stool samples were also collected at day 1, 2, 3, 4, 7, and 14 after inoculation from mice that were exposed to HEV *via* oral gavage. Viral RNA could be detected at single time points within the first week in some of the inoculated mice. However, HEV RNA could no longer be detected in the faecal samples collected at later time points (Figure 3), nor in the organs collected from these mice at day 56 (data not shown).

### Induction of Anti-Hepatitis E Virus ORF2 IgG Antibodies

**FIGURE 4:**
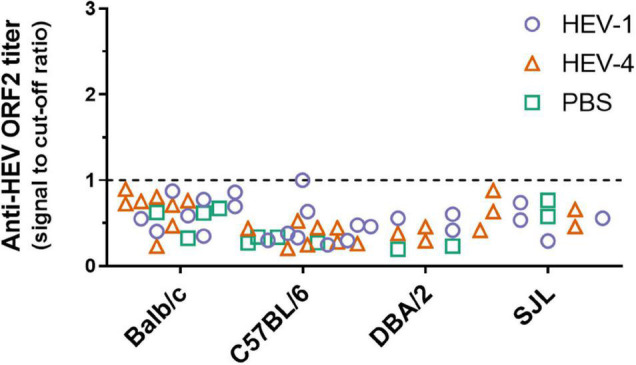
No anti-HEV ORF2 IgG antibodies could be detected in non-humanised mice at ten weeks post-inoculation with HEV-4 strain BeSW67HEV4-2008 or HEV-1 strain SAR-55. Plasma samples were collected ten weeks post-inoculation from Balb/c, C57BL/6, DBA/2, and SJL mice and anti-HEV ORF2 ELISA was performed to detect the presence of anti-HEV ORF2 IgG antibodies. Values are depicted as signal to cut-off ratios, where values above one are considered positive (above dashed line). PBS, phosphate buffered saline.

Anti-HEV ORF2 ELISA was performed to detect the potential presence of anti-HEV antibodies in immunocompetent mice, which could have been induced upon inoculation with HEV-1 or HEV-4. The assay was performed on plasma samples collected at week 10 after inoculation, but all mice scored negative (Figure 4).

### Phylogenetic Analysis

**FIGURE 5:**
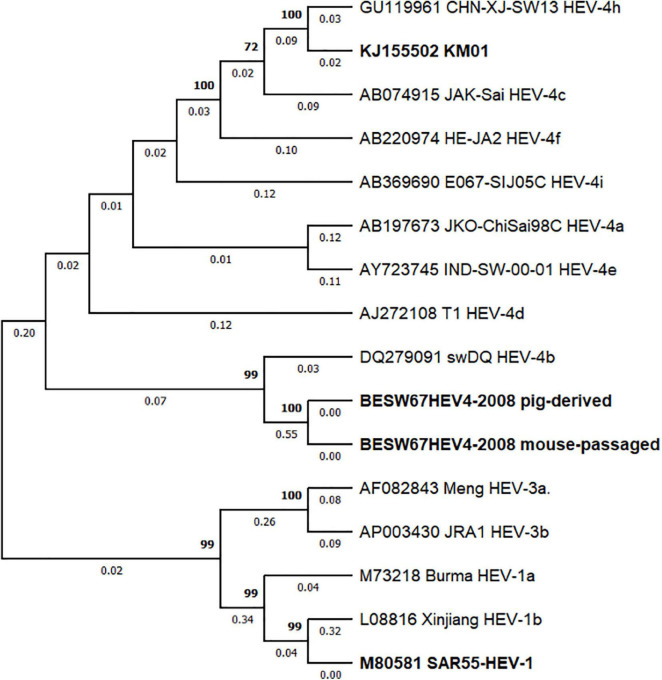
Phylogenetic analysis of HEV-4 strains BeSW67HEV4-2008 and KM01. A maximum likelihood phylogenetic tree was constructed with BeSW67HEV4-2008 (pig-derived and mouse-passaged), KM01 and 13 HEV reference sequences (Smith et al., 2020) based on the GTR + G + I model with 1,000 bootstrap replicas. Sequences of HEV-1 and HEV-3 were used as an outgroup to root the tree. Bootstrap values above 70% are depicted above the branches, whereas branch lengths are displayed under each branch. Sequence identifiers consist of GenBank accession numbers followed by strain name and subtype. HEV strains used in this research and KM01 are highlighted in bold.

In order to assess why a difference in infectability is seen between BeSW67HEV4-2008 and KM01, a phylogenetic tree was constructed based on the sequence of these isolates, and 12 reference sequences of HEV retrieved from Smith et al. (2020). This analysis confirmed that KM01 belongs to the HEV-4h subtype and the BeSW67HEV4-2008 viral strain belongs to HEV-4b (Figure 5).

## Discussion

The lack of an efficient small animal model for HEV has hampered the study of its replication and pathogenesis; and the discovery and development of specific anti-HEV drugs. Since HEV becomes more prevalent and more strains that are infectious to humans are being discovered, a suitable small animal model would be highly desirable. HEV infection in human liver-chimeric mice has already been intensively studied and these models can be used in the study of viral pathogenesis as well as the identification and preclinical evaluation of novel antiviral compounds. However, to date only HEV-1 and HEV-3 have been studied in these models.

Here, we describe effective HEV-4 infection in the humanised uPA^+/+^-SCID model with the viral strain BeSW67HEV4-2008. Experiments with the original pig-derived HEV-4 viral suspension did not result in robust infection as only three out of nine animals became infected (Figure 1). Of note is the low viral titer of the pig-derived HEV-4 stock (10^4^ IU/ml), which limited the amount of virus that could be injected into each mouse and contributed to the inefficient infection rate in this experiment. The onset of infection is only observed after 10 to 14 weeks post-inoculation. This slow course may be due to the low amount of virus injected or reflect the fact that this pig isolate may not replicate as efficiently in a human environment. Regardless of the late onset of infection, one mouse reached a faecal titer as high as 2 × 10^8^ IU/ml. This faecal suspension allowed the administration of a much higher viral dose and was used in subsequent experiments in humanised and non-humanised mice.

Humanised mice could be readily infected *via* intrasplenic or intraperitoneal injection, leading to active replication of the virus, as evidenced by the increase of HEV RNA in stool over time (Figure 2). Here, viral RNA was detected in faeces as soon as one week (intrasplenic injection) or two weeks (intraperitoneal injection) after inoculation. Overall, plasma HEV titers were consistently lower compared to faecal titers. This has previously been observed in this uPA^+/+^-SCID model for HEV-1 and HEV-3; as well as in chimpanzees, cynomolgus macaques, pigs, and humans (Tsarev et al., 1994a; Williams et al., 2001; Li et al., 2006; Sayed et al., 2017b). Furthermore, in stool, HEV RNA could be detected sooner than in plasma, which is in line with previous studies in human liver-chimeric mice and chimpanzees (Li et al., 2006; Sayed et al., 2017b). Human hepatocytes preferentially secrete HEV apically, directing the virus toward the bile and intestinal tract, compared to the secretion into the blood stream (Capelli et al., 2019). This imbalance probably results in faster detection of HEV in stool than plasma of infected animals as well as humans. Recently, HEV replication was shown in human primary intestinal cells. Here, preferred apical release of HEV was also reported (Marion et al., 2020), which may contribute to faster detection of HEV in faeces. However, in the human liver-chimeric mouse model, the intestines are not humanised and HEV was administered through intrasplenic and intraperitoneal injection, bypassing the intestines during infection. Therefore, secretion by intestinal cells does not contribute to the viral titer seen in faeces of these mice. Moreover, inoculation of human liver-chimeric mice through oral gavage did not result in HEV infection (Sayed et al., 2017b). Also, faecal and plasma titers of HEV-4 were significantly lower than HEV-1 titers, but were comparable to HEV-3 infection in the same model (Sayed et al., 2017b). This difference in infectivity reflects the clinical presentation in humans, where HEV-1 is more virulent compared to HEV-3 and HEV-4. Although often self-limiting, HEV-1 can cause an acute infection in immunocompetent individuals. On the contrary, HEV-3 or HEV-4 infections are mostly asymptomatic and seen in immunodeficient patients (Kamar et al., 2014). Infection of humanised mice with mouse-passaged virus resulted in earlier onset and higher titers compared to infections with the pig-derived stool suspension. The former were achieved by a 1.000-fold higher dose, which could explain the accelerated progression of the infection. However, viral adaptation to the human environment could also aid in the improved infection. Therefore, viral genomes of the pig-derived and the mouse-passaged stool suspensions were sequenced to reveal any adaptations. One point mutation (T3155C) was observed in the ORF1 DNA sequence, which did not result in an amended amino acid sequence.

Overall, we can conclude that human liver-chimeric mice are susceptible to HEV-4 infection. Further investigation is needed to reveal all aspects of HEV-4 infection and especially where the infection differs from HEV-1 and HEV-3 infections. This knowledge can shed light on the different pathogenesis between viral strains and aid in the discovery of specific therapies. Furthermore, the human liver-chimeric mouse models can be used to identify new HEV strains, infectious to humans. For example, two recent cases of infections in humans with rat HEV-C1 have been described, although this HEV strain is divergent from human HEV strains (Sridhar et al., 2018; Andonov et al., 2019). This will help to define potential sources of infection and hence specific precautions can be taken to lower or eliminate the risk of transmission to humans.

Although, the human liver-chimeric mouse models have greatly contributed to our understanding of the HEV virus, its lack of an adaptive immune system did not allow to fill a gap in our knowledge, which is the interplay between the virus and the host immune system upon infection. Thus, investigation into regular, immunocompetent mice as a model for HEV infection continues. Recent studies have demonstrated the susceptibility of regular and nude Balb/c mice to HEV-4 infection. However, these studies only studied one viral strain in detail (KM01). Here, we investigated a different HEV-4 strain (BeSW67HEV4-2008) for its ability to infect Balb/c mice as well as other regular and immunodeficient mouse strains.

Even though, the viral isolate can readily infect humanised mice *via* intrasplenic and intraperitoneal injection, we were unable to establish an infection in Balb/c, C57BL/6 or SCID mice *via* the same inoculation routes, as evidenced by the lack of HEV RNA in the stool, liver and other organs of these animals. Li et al. (2020) observed a difference in susceptibility to HEV-4 infection between Balb/c and C57BL/6 mice. This discrepancy may potentially be related to a difference in the immune response upon infection. C57BL/6 mice have a predominant Th1 immune response which is important in the clearance of intracellular pathogens, whereas Balb/c mice tend toward a Th2 response, commonly associated with responses against parasitic infections (Sellers et al., 2012). This may explain why C57BL/6 mice are able to clear the virus before onset of infection, as opposed to Balb/c mice. Similar to infection with KM01, C57BL/6 mice were not susceptible to HEV infection with the BeSW67HEV4-2008 viral strain. To further investigate this discrepancy, we expanded the examined mouse strains with DBA/2 and SJL mice. The DBA/2 mouse strain favours a Th2 response similar to Balb/c mice and SJL mice have an inverted B- to T-cell ratios compared to C57BL/6 mice. However, neither was susceptible to HEV-1 or HEV-4 infection. Therefore, to date, HEV-4 infection in non-humanised mice has only been described in Balb/c mice in combination with strain KM01. Because of extensive breeding at different facilities around the world, different substrains of Balb/c mice have been established. To assess if there is a difference in susceptibility between Balb/c substrains, HEV infection was compared in two substrains of Balb/c, available for purchase from Janvier Labs (France), Balb/cAnNRj and Balb/cJRj. Unfortunately, HEV-4 infection could not be established in either substrain.

Inoculation through oral gavage mimics best the natural faecal-oral transmission route. While HEV RNA could be detected at isolated time points days after inoculation, probably linked to input viral particles and a confirmation of correct administration rather than virus replication, no HEV RNA was detected at week 2, 4, or 8 (Figure 3). These results are supported by the finding of Sayed et al. (2017b) who demonstrated human-liver uPA^+/+^-SCID mice could not be infected with HEV *via* oral gavage. Furthermore, other studies in cynomolgus macaques and pigs reported similar findings (Tsarev et al., 1994b; Kasorndorkbua et al., 2002, 2004), suggesting certain essential (human) receptors may be lacking or are different in the mouse intestine.

There certainly remains the possibility that the virus is rapidly cleared before a robust infection can be established. In that case, one would expect to find anti-HEV IgG antibodies. Plasma, collected at week 10, of all immunocompetent mouse strains was subjected to anti-HEV ORF2 ELISA. However, antibody levels were all below the cut-off of the assay (Figure 4). One mouse showed a borderline level of anti-HEV antibodies, but at week 8 the antibody levels were clearly negative for this mouse (signal to cut-off = 0.329). Since all mice were sacrificed at week 10 post-inoculation, no further follow-up of the anti-HEV antibody levels could be performed. These results are in line with findings of Li et al. (2020) where no elevation in IgG or IgM antibodies was detected in Balb/c or C57BL/6 mice up to four weeks post-inoculation.

In conclusion, we were unable to corroborate the results published by Li et al. (2020) with our HEV-4 strain BeSW67HEV4-2008. Phylogenetic analysis of both viral strains revealed that the BeSW67HEV4-2008 strain is part of the HEV-4b subtype, whereas KM01 and other strains, shown to infect mice are of the HEV-4h subtype (Figure 5), indicating mice may not be susceptible to infections with all HEV-4 subtypes. For further assessment of this theory, more HEV-4 strains need to be tested for their ability to infect (non-humanised) mice and this may finally lead to a new immunocompetent HEV mouse model allowing extensive study of HEV immunity and pathogenesis.

## Data Availability Statement

The data presented in the study are deposited in the GenBank repository, accession number OM388298.

## Ethics Statement

The animal study was reviewed and approved by the Animal Ethics Committee of the Faculty of Medicine and Health Sciences of Ghent University.

## Author Contributions

LC performed experiments, analysed data, and wrote the manuscript. LV performed experiments. RH-VdH and WHMVdP provided essential reagents and critically reviewed the manuscript. PM conceptualised the project, analysed data, and critically reviewed the manuscript. All authors contributed to the article and approved the submitted version.

## Conflict of Interest

The authors declare that the research was conducted in the absence of any commercial or financial relationships that could be construed as a potential conflict of interest.

## Publisher’s Note

All claims expressed in this article are solely those of the authors and do not necessarily represent those of their affiliated organizations, or those of the publisher, the editors and the reviewers. Any product that may be evaluated in this article, or claim that may be made by its manufacturer, is not guaranteed or endorsed by the publisher.
